# Integrated 3D Information for Custom-Made Bone Grafts: Focus on Biphasic Calcium Phosphate Bone Substitute Biomaterials

**DOI:** 10.3390/ijerph17144931

**Published:** 2020-07-08

**Authors:** Alessandra Giuliani, Maria Laura Gatto, Luigi Gobbi, Francesco Guido Mangano, Carlo Mangano

**Affiliations:** 1 Department of Clinical Science, Polytechnic University of Marche, Via Brecce Bianche, 60131 Ancona, Italy; 2 Department of Materials, Environmental Sciences and Urban Planning, Polytechnic University of Marche, Via Brecce Bianche, 60131 Ancona, Italy; m.l.gatto@pm.univpm.it (M.L.G.); l.gobbi@univpm.it (L.G.); 3 Department of Prevention and Communal Dentistry, Sechenov First Moscow State Medical University, 119991 Moscow, Russia; francescoguidomangano@gmail.com; 4 Private Practice, 22015 Gravedona, CO, Italy; camangan@gmail.com

**Keywords:** bone grafts, biphasic calcium phosphate, 3D information, morphometric analysis, mechanical analysis, X-ray microtomography

## Abstract

Purpose: Several studies showed that the sintering temperature of 1250 °C could affect the formation of α-Ca_3_(PO_4_)_2_, which is responsible for the reduction of the hardness value of biphasic calcium phosphate biocomposites, but they did not evaluate the inference of the sintering time at peak temperature on transition of β-Ca_3_(PO_4_)_2_ to α-Ca_3_(PO_4_)_2_. This analysis explored, in an innovative way, inferences and correlations between volumetric microstructure, mechanical properties, sintering temperature, and time at peak temperature in order to find the best sintering conditions for biphasic calcium phosphate composites grafted in severe alveolar bone defects. Methods: Sintered biphasic calcium phosphates (30%-hydroxyapatite/70%-tricalcium phosphate) were tested by microCT imaging for the 3D morphometric analysis, by compressive loading to find their mechanical parameters, and by X-ray diffraction to quantify the phases via Rietveld refinement for different sintering temperatures and times at the peak temperature. Data were analysed in terms of statistical inference using Pearson’s correlation coefficients. Results: All the studied scaffolds closely mimicked the alveolar organization of the jawbone, independently on the sintering temperatures and times; however, mechanical testing revealed that the group with peak temperature, which lasted for 2 hours at 1250 °C, showed the highest strength both at the ultimate point and at fracture point. Conclusion: The good mechanical performances of the group with peak temperature, which lasted for 2 hours at 1250 °C, is most likely due to the absence of the α-Ca_3_(PO_4_)_2_ phase, as revealed by X-ray diffraction. However, we detected its presence after sintering at the same peak temperature for longer times, showing the time-dependence, combined with the temperature-dependence, of the β-Ca_3_(PO_4_)_2_ to α-Ca_3_(PO_4_)_2_ transition.

## 1. Introduction

Dental implants were demonstrated to have long survival times, especially in mandibles [1,2]. However, successful implantation can be achieved only when a sufficient jawbone volume is present [2,3,4]; if diseases, trauma, cysts, or tumors occur, they often produce severe alveolar bone defects, suggesting alveolar ridge augmentation procedures before implantation [3,4,5].

Autografts and allografts have been shown to achieve varying degrees of success in restoring form and function, often still involving risks; for example, morbidity at the donor site is a common side effect of the autografts, while allografts sometimes cause disease transmission [6,7].

An ideal substitute biomaterial should be similar to the hosting bone; the major function of scaffolds is to balance temporary mechanical functions with mass transport to aid biological delivery and tissue regeneration. Scaffolds should be made of biocompatible materials and therefore never cause adverse immune responses in the host tissue after grafting. Furthermore, the degradation by-products of scaffolds must be non-cytotoxic.

A well-known potential group of scaffolds comprises bioceramics, which have been successfully used as bone substitute biomaterials (BSBs) in clinical applications for many years. They can be produced, in customized ways, with optimized 3D shapes, porosity, and topographies. Indeed, most of them are obtained at a high temperature (>1000 °C) by sintering reactions. These biomaterials include β-tricalcium phosphate (β-TCP; β-Ca_3_(PO_4_)_2_), sintered hydroxyapatite (HA; °Ca_10_(PO_4_)_6_(OH)_2_), or their composites called biphasic calcium phosphates (BCPs). Morphologically, porous BSBs have been designed to mimic the microstructure of trabecular bone. Indeed, a porous structure is light in weight and provides appropriate space for the ingrowth of the bone tissue, partially achieving the replacement of the biomaterial by newly formed bone [8,9].

Scaffolds act as temporary extracellular matrixes, assisting proliferation, differentiation, and biosynthesis of cells on their surface and pores. An adequate surface roughness is necessary to achieve cell fixation; moreover, scaffold morphometry plays a relevant role in order to achieve bone ingrowth. Indeed, the combination of high porosity, adequate pore dimensions, and interconnectivity are important parameters for scaffold fabrication [10,11,12] because they allow cell migration, vascularization, as well as diffusion of nutrients. Macroporosity (pores of 100–500 µm in diameter) is thought to contribute to osteogenesis by facilitating cell and ion transport, while microporosity (pores with diameter <100 µm) improves bone growth into scaffolds by increasing surface area for protein adsorption, increasing ionic solubility in the microenvironment, and providing attachment points for osteoblasts. Moreover, interconnectivity of pores provides spacing for the vasculature required for new bone formation.

Mechanical properties of scaffolds also play another important role in bone engineering. Important mechanical indices of bone include Young’s modulus, toughness, shear modulus, tensile strength, fatigue strength, and compressive strength; mimicking the properties of hosting bone at a macroscopic level is of paramount importance for a bone scaffold during the implantation stage, sustaining the regeneration of new tissue. From a mechanical point of view, calcium phosphate bioceramics appear to be brittle polycrystalline materials; their mechanical properties are governed by crystallinity, grain size, grain boundaries, porosity, composition, presence of impurities, and manufacturing processes. Bending, compressive, and tensile strengths of porous BCP scaffolds are generally: 2–11 MPa, 2–100 MPa, and 3 MPa, respectively. Scaffold strength is strongly influenced by molecular balancing, increasing with Ca/P ratio up to a maximum value of around 1.67 and decreasing suddenly after when Ca/P > 1.67. Moreover, it was shown that strength decreases almost exponentially with increasing porosity [13].

When producing BCPs by sintering, different process regimes allow the modification of scaffold performances in customized ways, once grafted in a patient bone defect. In particular, modulation of sintering temperature was shown to change the BCP biocomposite characteristics (phase’s formation, porosity, and hardness properties) [14], also using mathematical and digital models [15,16]. Sintering temperature was shown to influence the formation of α-tricalcium phosphate (α-TCP; α-Ca_3_(PO_4_)_2_), affect the 3D porosity of the samples [14,16], and possibly reduce the biomaterial hardness, consequently affecting performance in patients. In particular, it was shown that despite an ideal porosity, there is no bone growth on α-TCP scaffolds because of its rapid dissolution, making the environment near the surface of the biomaterial more acidic [17].

Thus, phase composition, porosity amount and distribution at a 3D level, and mechanical properties were shown to be correlated in BCPs [18,19]. In particular, BCPs with the composition of 30% HA-70% TCP were investigated in the temperature range between 1000 °C and 1300 °C [19]; data showed that consolidation, grain growth, and Vickers hardness generally increase with sintering temperature up to 1200 °C. However, cracks could occur after sintering at 1200 °C. Moreover, the same study showed that the highest compressive strength, modulus of elasticity, and toughness are achieved with sintering at 1100 °C, they decay with sintering at 1200 °C, and increase again with sintering above 1300 °C. However, the problem to find the best sintering conditions is not trivial and deserves further research: indeed, the creation of optimized processes, with correct sequences and times, could limit unwanted phase transition from β-Ca_3_(PO_4_)_2_ to α-Ca_3_(PO_4_)_2_ [15], despite the use of high sintering temperatures [14].

In this context, several computer-aided imaging techniques were used to investigate the microarchitecture of BSBs; in particular, the gold standard for a reliable 3D examination is based on high-resolution X-ray computed tomography (microCT). MicroCT was not only successfully used to reconstruct the morphometry of bone tissue at different length-scales [20,21,22,23,24] and in different environmental and genetic contexts [25,26,27,28], but it is nowadays the elective technique for the characterization of biomaterials used in bone tissue engineering [29] and also in dental districts [30,31,32,33,34,35].

We recently performed a first successful microCT test, combining morphometric 3D analysis with a new protocol of study referred to as BCP’s mechanical properties [30]; in the present study, BCP were studied again during compressive tests up to fracture, testing, for the first time to the authors’ knowledge, inferences and correlations between 3D microstructure, mechanical properties, sintering temperature, and time at peak temperature. The objective was the monitoring of β-Ca_3_(PO_4_)_2_ to α-Ca_3_(PO_4_)_2_ transition, finding the best processing conditions for biphasic calcium phosphate biocomposites.

## 2. Materials and Methods

Biphasic calcium phosphate (30% of HA and 70% of TCP) samples were produced by sintering of powders using two different protocols: in both of them, the first plateau of temperature reached 800 °C for 2 hours, while in the second plateau, the peak temperatures were different, namely T = 1200 °C and T = 1250 °C. Both peak temperatures samples were sintered for 2, 4, or 8 hours; afterwards, they were tested, first by microCT to get the 3D morphometric analysis and then by loading tests in continuous mode, with compression up to fracture to obtain the reference points in the stress/strain curves.

### 2.1. Biomaterials

Biphasic calcium phosphate samples (30%HA/70%TCP) were obtained by synthesis in the laboratory of the Biotec srl, Povolaro, Italy. Six groups of samples were produced:

- T = 1200 °C@2h: nr.3 samples at peak sintering temperature of 1200 °C for 2 hours;
- T = 1200 °C@4h: nr.3 samples at peak sintering temperature of 1200 °C for 4 hours;
- T = 1200 °C@8h: nr.3 samples at peak sintering temperature of 1200 °C for 8 hours;
- T = 1250 °C@2h: nr.3 samples at peak sintering temperature of 1250 °C for 2 hours;
- T = 1250 °C@4h: nr.3 samples at peak sintering temperature of 1250 °C for 4 hours;
- T = 1250 °C@8h: nr.3 samples at peak sintering temperature of 1250 °C for 8 hours.

All the scaffolds were small blocks with a volume of about 125 mm³.

### 2.2. High-Resolution Tomography (microCT)

A Skyscan 1174 tomograph (SkyScan-Bruker, Antwerp, Belgium) was used to perform the microCT analysis using the following experimental conditions: voltage: 50 kV; current: 800 μA; pixel size: 9.5 μm; rotation step: 0.2° for 180°; exposure time per projection: 10 s; filter for the optimization of X-ray photon energy and transmission through samples: 1 mm of Al.

We obtained 8-bit (TIFF) projections that were processed by the NRecon software (SkyScan-Bruker, Antwerp, Belgium), obtaining stacks of cross-sectional slices. The slices consisted of 8-bit (BMP) images, generated with the following settings: smoothing = 4; ring artefacts reduction = 2; beam hardening correction = 10.

The 3D reconstructions were realized using the VG Studio MAX 1.2 software (Volume Graphics, Heidelberg, Germany). Two different peaks were observed in the grey level histogram, one representing the air and the other the biphasic calcium phosphate scaffold. They were separated by the implementation of the Mixture Modeling algorithm (NIH ImageJ Plugin).

Afterwards, we measured the following quantitative morphometric indices [36]: Sample Surface to Sample Volume ratio—SS/SV (mm^-1^), Sample Volume to Total Volume ratio—SV/TV (%), mean Strut Thickness—S.Th. (µm), mean Strut Number—S.Nr. (mm^-1^), mean Porosity (%), and mean Strut Spacing—S.Sp. (µm).

Moreover, data on the eventual presence of preferential orientations were also extracted, introducing the anisotropy degree index (DA), which varies between 0, corresponding to the perfect isotropy of the scaffold structure, and 1, representing structures perfectly oriented in agreement with a single plane or axis. The last calculated parameter was the pore connectivity density—P.Conn.D (pixel^−3^), which gives higher values for better-connected pores and lower values for poorly connected ones.

### 2.3. Compressive Testing

Compressive tests, in continuum mode up to fracture, were performed in all the scaffolds, using a Material Testing Stage (MTS1) implemented within the Bruker SkyScan 1174 experimental hall, in place of the standard sample holder. During the tests, the scaffolds were submitted to loading by a dedicated software; the representing loading curve was displayed on the screen in real time, with resulting deformation measured by a precision displacement sensor.

The MST1 stage has the following characteristics: maximum force: 440 N; displacement sensor accuracy: ± 0.01 mm; load measurement accuracy = ± 4 N (± 1% of the full range); maximum object diameter: 20 mm; maximum object height (in compression mode): 23 mm.

### 2.4. X-ray Diffraction

All samples were also analysed using X-ray diffraction (XRD- Bruker D8 Advance, Cu-Kα-Ni and digitally filtered) after sintering. For the XRD measurements, a 2θ scan range of 4–60° with 0.012° steps and 0.15 s counts per step was set. A Rietveld refinement package (ISO 13779-3:2018) was used to quantify the different crystallographic phases. The XRD analyses were performed at the RMS Foundation (Beltlach, CH).

### 2.5. Statistical Analysis

All values were expressed as mean and standard deviations. The One Way Analysis of Variance tests, with All Pairwise Multiple Comparison Procedures performed by the Holm-Sidak method (SigmaStat software 3.5, Systat, San Jose, CA, USA), were used for the statistical analysis of the morphometric microCT data. The statistical significance was analyzed at three levels: *p* < 0.05, *p* < 0.01, *p* < 0.001.

The Pearson correlation coefficient (*PCC*), also referred to as Pearson’s *r*, was also evaluated to measure the linear correlation between two variables, X and Y, randomly selected between the microarchitecture structural indices and relevant points of the obtained Stress vs Strain curves. The software that was used for the PCC evaluation is freely available at the Alcula.com site (accessed on 30 April 2020).

## 3. Results

All the scaffolds had the same biphasic composition: 30% HA and 70% TCP. This balancing of phases was previously demonstrated to be good in preserving the volumes during grafting because of HA slow resorption and TCP rapid resorption favoring bone regeneration [32,33].

The investigation was focused on the analysis of microarchitecture, strength to compressive forces, and inference between these indices. In particular, possible correlations between these indices, sintering time, and temperature conditions were considered.

### 3.1. MicroCT Analysis

**Figure 1:**
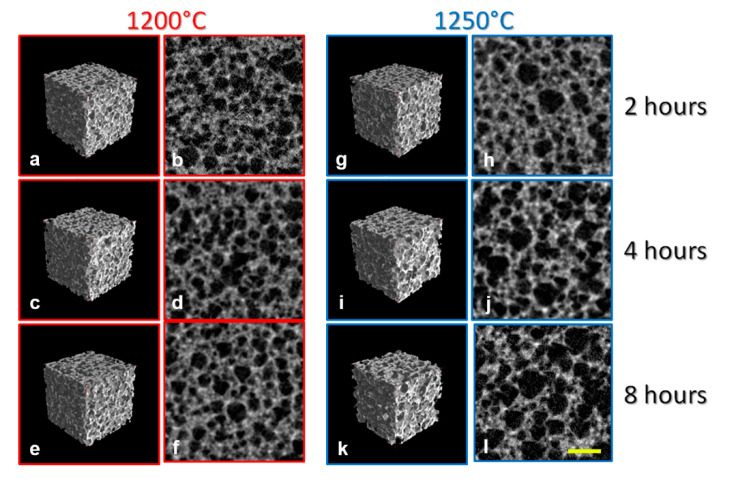
MicroCT analysis of the sintered biomaterials: (**a**–**f**) Samples sintered at the peak temperature of 1200 °C; (**g**–**l**) Samples sintered at the peak temperature of 1250 °C. 3D reconstructions (**a**,**c**,**e**,**g**,**i**,**k**) and axial (**b**,**d**,**f**,**j**,**l**) 2D sections before compressive loading; representative sample of: (**a,b**) T = 1200 °C@2 h group; (**c**,**d**) T = 1200°C@4 h group; (**e**,**f**) T = 1200 °C@8 h group; (**g**,**h**) T = 1250 °C@2 h group; (**i**,**j**) T = 1250 °C@4 h group; (**k**,**l**) T = 1250 °C@8 h group. Larger pores for samples sintered at T = 1250 °C with respect to those sintered at T = 1200 °C were found, independently from sintering time at the peak temperature. Yellow bar: 500 µm.

The microCT experiments were performed using a SkyScan-Bruker 1174 microCT system, equipped with a Material Testing Stage (MTS1), installed at the CISMIN Centre of the Polytechnic University of Marche (Ancona, Italy). MicroCT images of representative samples for each group of the study are reported in Figure 1: in particular, samples sintered at the peak temperature of 1200 °C for 2 hours (Figure 1a,b), samples sintered at the peak temperature of 1200 °C for 4 hours (Figure 1c,d), samples sintered at the peak temperature of 1200 °C for 8 hours (Figure 1e,f), samples sintered at the peak temperature of 1250 °C for 2 hours (Figure 1g,h), samples sintered at the peak temperature of 1250 °C for 4 hours (Figure 1i,j), and samples sintered at the peak temperature of 1250 °C for 8 hours (Figure 1k,l) were shown. At the peak temperature of 2 hours, larger pores for the group at T = 1250 °C with respect to the group at T = 1200 °C were found. No other evident morphometric mismatches between groups of the study were found from the simple visual comparison of the 3D reconstructions.

**Figure 2:**
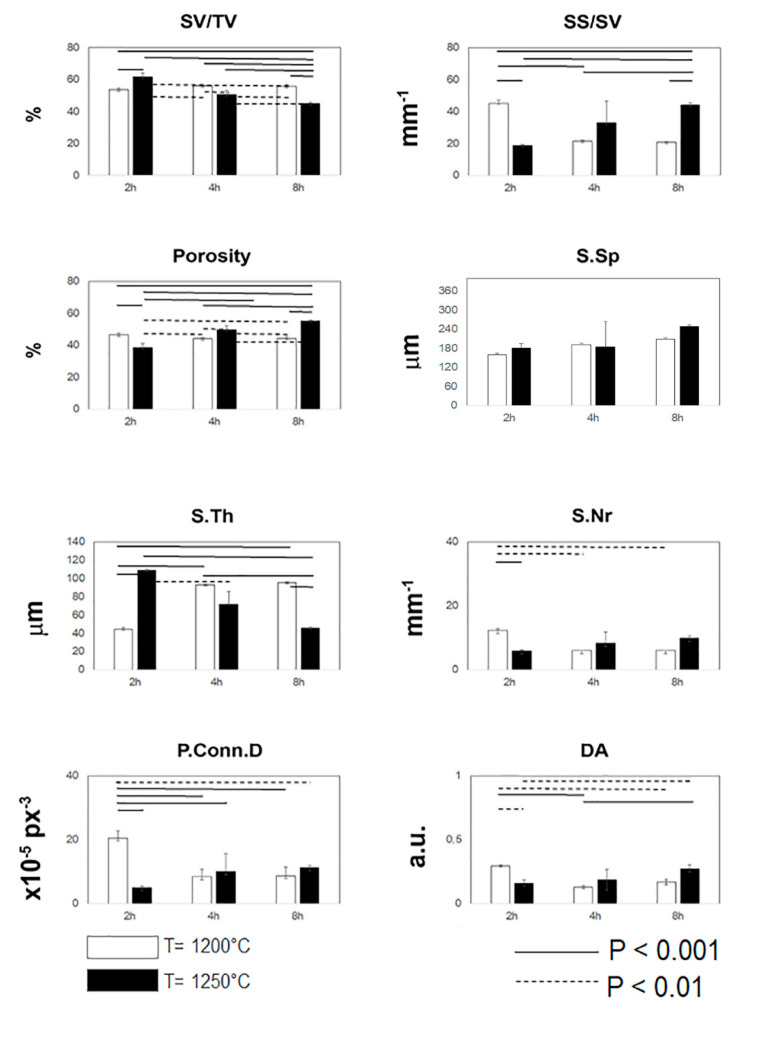
Morphometric 3D data, as measured in the different samples before compressive tests by microCT. Histograms graphically depict morphometric parameters as a function of the selected groups of samples. Mean and standard deviation data are indicated for each group of the study. Statistically significant differences between groups are highlighted (dashed line: *p* < 0.01; solid line: *p* < 0.001).

Thus, in order to get reliable information on the microarchitecture, the quantitative morphometric analysis of the complete set of samples was carried out. These data were reported in Figure 2. The characterization of the 3D mineralized microarchitecture showed that at the peak temperature of 1200°C and at increasing times of sintering, several parameters remained constant. We observed, as a trend, an increasing of S.Th. and S.Sp. and a slight decreasing of SS/SV, S.Nr, and P.Conn.D. Conversely, at the peak temperature of 1250 °C and at increasing times of sintering, the variability was much wider; indeed, while SV/TV and S.Th. decreased, the remaining indices increased at increasing times of permanence at the peak temperature. Interestingly, in the case of the group maintained for 2 hours at the peak temperature of 1200 °C, the structure was made of much more thin trabeculae with pores highly connected. However, the too small mean size of the pores (S.Sp) advises against the use of these sintering parameters.

With the exclusion of the previous group, beyond other significant deviations between corresponding data at the peak temperature of T = 1200 °C and T = 1250 °C, which were statistically detected and are reported in Figure 2 for all the indices, we found a high standard deviation for almost all the indices detected in the group maintained for 4 hours at the peak temperature of 1250 °C.

### 3.2. Mechanical Characterization

The strength of the scaffolds was investigated by compressive tests in the axial direction and in continuous mode up to fracture. This study is of fundamental interest: indeed, compressive forces produced during the implant insertion and afterwards, during mastication, cause a modification of the biomaterial performance and of the jaw homeostasis. When loading exceeds the resistance limit of the biomaterial, the osteoblasts start to reduce their activity, with the consequent bone resorption.

Stress (σ) and strain (ε) parameters were recorded, classically defined as below:

σ= F/A_0_ (1)

where F is the compressive force perpendicularly acting on the cross section A_0_ of the sample and

ε = |(l_i_-l_0_)/l_0_| (2)

where l_0_ is the length of the sample before loading and l_i_ is its length during testing.

The length of sample l_0_ was precisely determined by X-ray projections before the mechanical test of each sample. Moreover, the cross section A_0_ of each sample was found, by microCT morphometric analysis of the first 10 slices of contact with the loading piston of the MTS1 device, taking into account the actual contact surface, i.e., subtracting the calculated porosity from the overall area. Indeed, samples produced by sintering normally have a homogeneous porosity throughout their thickness. Thus, we arbitrarily decided to use the first 10 contact slices to have a representative situation on 100 µm of thickness, which is a reasonable volume to find A_0_.

The stress–strain profiles of the studied scaffolds allowed us to get the data shown in Table 1. While there is not a clear trend depending on the time of permanence at the peak temperature for samples of groups with T = 1200 °C, for samples at T = 1250 °C, as the duration of the treatment increases, yield, ultimate, and fracture stresses seemed to decrease. At T = 1250 °C, the samples with peak temperature lasting for 8 hours have values comparable to those at 1200 °C, with higher sample strains both at the ultimate point (UP) and at fracture point (FP).

### 3.3. Statistical Inference

**Figure 3:**
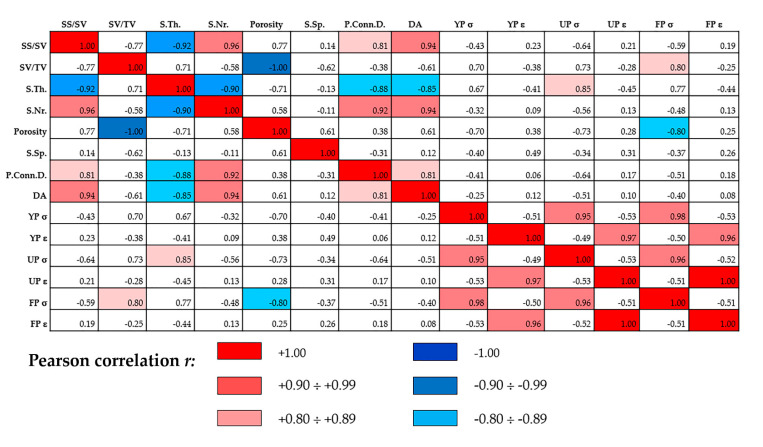
Pearson’s correlation coefficients of quantitative microCT and compressive test parameters. YP = Yield Point; UP = Ultimate Point; FP = Fracture Point.

The Pearson’s correlation coefficients (PCC) between indexes extracted from the overall analysis are summarized in Figure 3. Statistical inference based on PCC was focused on testing the null hypothesis that a true correlation does not exist between two indexes extracted from the microarchitecture analysis and/or from compressive testing, based on the value of the sample correlation coefficient *r*.

As *r* = 0.8 may not be an optimal value when verifying a possible correlation using high-quality devices like 3D high-resolution microCT and compressive testing equipment like MST1, we discriminated good and strong (direct/inverse) correlations when obtaining 0.80 ≤ |*r*| ≤ 0.89 and 0.90 ≤ |*r*| ≤ 0.99, respectively.

Strong direct correlations were found between specific surface and number of struts, specific surface and anisotropy degree, number of struts and pore connectivity density, and number of struts and anisotropy degree. Obviously, strong direct correlations were also found between the stress values in the yield, ultimate and fracture points, and between the strain values in the same points. Conversely, strong inverse correlations were found between specific surface and struts mean thickness, number of struts, and struts mean thickness. Good direct correlations were found between specific surface and pore connectivity density and this last parameter with the anisotropy degree, but interestingly, also between specific volume and stress at the fracture point and between struts mean thickness and stress at the ultimate point. Moreover, good inverse correlations were found between struts mean thickness and pore connectivity density, struts mean thickness and anisotropy degree, and interestingly, also between the overall porosity and stress at the fracture point.

### 3.4. X-ray Diffraction Analysis

X-ray diffraction (XRD) measurements of the BCPs revealed, for samples sintered at the peak temperature of 1200 °C and 1250 °C for 2 hours, not appreciable quantitatives of α-TCP and tetracalcium phosphate (TTCP) below the minimum detection limit (Table 2). Conversely, if sintering at the peak temperature of 1250 °C is prolonged for 4 hours, significant amounts of α-TCP appear. Indeed, we showed that in this condition, the composition is far away from the target 30%HA/70%TCP, with significantly lower amounts of β-TCP (around 50%) and with α-TCP contents above 19%.

## 4. Discussion

The recent literature widely reports successful bone regeneration using biphasic calcium phosphate scaffolds, with special reference to dental districts [30,37,38,39,40,41,42,43,44,45,46]. However, just a single study of ours described an integrated morphological, morphometric, and mechanical 3D analysis on these biomaterials [30].

In the present study, blocks of biphasic calcium phosphate scaffolds, produced by sintering at the two critical peak temperatures of 1200 °C and 1250 °C, were studied by an innovative integrated approach based on microCT morphometric 3D analysis, mechanical testing, and X-ray diffraction.

In general, all the studied scaffolds closely mimicked the alveolar organization of jawbone, having high medullar spaces interconnectivity. The mean size of the macropores (S.Sp. index) was found to be well above 100 μm in all the groups of study; this dimension is considered the recommended minimum size for bone tissue engineering because it allows bone cells migration and colonization into the scaffold, its vascularisation [47], and the consequent osteointegration of the scaffold in the jaw [33,48]. Specifically, the morphometric analysis by microCT showed that for the 3D mineralized microarchitecture of the scaffolds produced at the peak temperature of 1200 °C, several structural indices remained constant at increasing times of sintering. Conversely, we observed a much wider variability at the peak temperature of 1250 °C, with decreased BCP volumes and thinner struts at increasing times of sintering. Moreover, we observed that overall porosity and pore interconnectivity decreased for 1200 °C but increased for 1250 °C sintering over time. In this context, Bignon et al. [11] noted that BCP shows a total porosity, which decreases as a function of the temperature in the range between 1200°C and 1250 °C. The trend was similar to the profile achieved in our study, i.e., with a porosity that decreases up to 1250 °C and then grows more and more according to the sintering time. Indeed, a general rule observed in several studies of bioceramics is that shorter times at higher sintering temperatures produce similar effects to longer times at lower sintering temperatures; obviously, up to now, this was a purely indicative equivalence, justifying the present study novelty. The same study [11] also showed that the micropores change in number and distribution as a function of temperature; i.e., by increasing the temperature but maintaining the same sintering time, the micropores tend to disappear. This could explain the trend of our data on pore interconnectivity.

Several physical forces and moments act daily on the jawbone during speaking or eating. In particular, during mastication, the jaw is subjected to compressive stress that is in non-linear relation with the strain [30] because of the yielding caused by debonding of osteons at cement lines and microfractures [49]. Compressive strengths of cancellous bone were found in the range of 2–12 MPa [50,51], depending on the skeletal site; thus, developing constructs as scaffolds with mechanical strength similar to the hosting bone is of paramount importance. Indeed, mechanical properties of BCP biomaterials have been studied for several years in order to increase their performance after grafting in jawbone [11,12,13,16,17,18,19]. The mechanical testing performed in the present study did not show a relevant variability depending on the time of permanence at the peak temperature for samples of groups at T = 1200 °C; conversely, for groups at T = 1250 °C, yield, ultimate, and fracture stresses seemed to decrease as the duration of the sintering increased. Specifically, the group with peak temperature lasting for 2 hours at 1250 °C showed the highest strength both at the ultimate point (UP) and at fracture point (FP). One more time, we should refer to the Bignon et al. [11] study in discussing our data: indeed, their compressive strength curve reflects our data, indicating that there is a critical point, which in our experiments, corresponds to T = 1250 °C for 2 hours, beyond which the material changes its mechanical properties. This is most likely due to the α-TCP formation, starting from the group lasting for 4 hours at T = 1250 °C.

In this context, the formation of α-TCP was shown to be responsible for the reduction of the hardness value of BCP biocomposites [16], hitherto making the use of temperatures above 1200 °C for sintering of scaffolds used for severe alveolar bone defects challenging. Nevertheless, while some authors showed that the key experimental variables to monitor during the allotropic α↔β-tricalcium phosphate phase transformations included the composition of the starting Ca/P ratio, the maximum heat treatment temperatures, and the cooling rates [52], no studies evaluated the inference of the sintering time at peak temperature, as we did in the present experiment. Indeed, our XRD data demonstrated the absence of the α-TCP in the group, with peak temperature lasting for 2 hours at 1250 °C, but its presence for sintering at the peak temperature of 1250 °C for longer times. Ca_3_(PO_4_)_2_ consists of three polymorphs: Kreidler and Hummel’s phase diagram [53] indicated that β-Ca_3_(PO_4_)_2_, which is rhombohedral, transforms into α-Ca_3_(PO_4_)_2_ upon heating at 1100-1200 °C; α-Ca_3_(PO_4_)_2_ is monoclinic and above 1400 °C, it converts into α’-Ca_3_(PO_4_)_2_, which is hexagonal. While α’-Ca_3_(PO_4_)_2_ is only stable above 1400 °C and reverts towards α-Ca_3_(PO_4_)_2_ immediately after cooling from such a temperature, α-Ca_3_(PO_4_)_2_ is metastable at room temperature and discussion about the cooling rate required to avoid reconversion to β-Ca_3_(PO_4_)_2_ is nowadays wide in the scientific community. Indeed, bioceramics based on α-Ca_3_(PO_4_)_2_ or containing the α-Ca_3_(PO_4_)_2_ are usually produced to obtain a calcium phosphate material with a higher dissolution rate [54,55]. However, very recent in-vitro studies on biocompatibility of biphasic TCP, with high contents of α- Ca_3_(PO_4_)_2_ , seeded with cells showed that in spite of the chemical similarity of α- Ca_3_(PO_4_)_2_ to the mineralized component of bone tissue, α-TCP was shown to cause the suppression of cell activity because of the environment’s acidification near the surface of the biomaterial [17]. Thus, as several authors argued that low cooling rates, after TCP sintering above 1200 °C, avoid the formation of β- Ca_3_(PO_4_)_2_ , it is likely to expect that fast sintering at peak temperature above 1200 °C may instead contribute to the exclusive presence of β-Ca_3_(PO_4_)_2_ at room temperature, like happened in our group of study, with peak temperature lasting for 2 hours at 1250 °C.

Moreover, in order to verify if relevant correlations exist between the 3D microarchitecture indices and the compressive strength, the statistical inference based on PCC was evaluated. Good direct correlations were found between the scaffold volume and strength at fracture point and between the strut mean thickness and the strength at the ultimate point. Interestingly, a good inverse correlation was also found between the overall 3D porosity and strength at the fracture point. This is in agreement with previous observations of other authors [16,18], observing inverse correlations between compressive strength and percentage of macroporosity or sintering temperature.

## 5. Conclusions

In order to describe the general integrity of a biomaterial in terms of fracture resistance, its strength is usually studied by mechanical testing together with its microarchitecture. From the morphometric analysis, it is expected that the biomaterial mimic the morphometry of the hosting bone site; from the compressive loading, it is expected to get a biomaterial with the same stiffness of the hosting healthy bone, withstanding strain during loading, but of which is also flexible and allows energy absorption during impact loading.

The BCPs investigated in this study were proved to have a morphometric structure similar to native jawbone, independently of whether they were sintered at different peak temperatures and for different times. However, we found that the group with peak temperature lasting for 2 hours at 1250 °C showed the highest strength, both at ultimate and at fracture point. As previously shown by other authors [14,15,16], the ultimate stress achieved by samples that were sintered at the peak temperature of 1250 °C for longer times was significantly lower. This was shown to be due, for times longer than 2 hours at peak temperatures above 1200 °C, to allotropic transformation of TCP from β to α phase, shown to occur by XRD; in fact, the α phase is brittle and with reverse transformation, is not totally achieved during cooling to room temperature [14,16].

## Figures and Tables

**Table 1:** Compressive Tests. Mean (std.dev) are listed.

Group	Yield Point (YP)		Ultimate Point (UP)		Fracture Point (FP)	
	Stress (MPa)	Strain (%)	Stress (MPa)	Strain (%)	Stress (MPa)	Strain (%)
**T = 1200 °C** **2 hours**	2.0 (0.6)	2.7 (1.9)	2.7 (0.1)	4.6 (2.6)	2.6 (0.8)	5.0 (2.8)
**T = 1200 °C** **4 hours**	1.7 (0.1)	4.1 (1.3)	2.9 (0.4)	6.1 (1.3)	2.5 (0.8)	6.6 (1.6)
**T = 1200 °C** **8 hours**	1.9 (0.2)	2.2 (0.4)	3.0 (0.4)	3.7 (0.4)	2.8 (0.2)	4.0 (0.3)
**T = 1250 °C** **2 hours**	3.2 (0.8)	2.3 (0.2)	4.4 (1.0)	3.7 (0.7)	4.0 (1.3)	4.0 (0.7)
**T = 1250 °C** **4 hours**	2.0 (0.8)	2.0 (0.3)	3.2 (0.6)	3.4 (1.1)	2.6 (0.3)	3.7 (1.2)
**T = 1250 °C** **8 hours**	1.7 (0.5)	4.0 (1.0)	2.6 (0.1)	5.4 (1.4)	2.3 (0.1)	5.7 (1.3)

**Table 2:** Quantitative evaluation of phases, as obtained by X-ray diffraction (XRD). Values are expressed as percentages (%). DL: detection limit.

Sample	HA	β-TCP	CaO	α-TCP	TTCP
**T = 1200 °C@2hours**	28.27	71.15	>0.04 and < 0.06	< DL	< DL
**T = 1250 °C@2hours**	28.51	70.77	>0.04 and < 0.06	< DL	< DL
**T = 1250 °C@4hours**	31.00	49.79	< DL	19.20	< DL

## Data Availability

The datasets are available from the author upon reasonable request.

## Abbreviations

BSB	Bone substitute biomaterial
3D	Three-dimensions
BCP	Biphasic calcium phosphate
MicroCT	Microtomography
PCC	Pearson correlation coefficient
XRD	X-ray diffraction
